# Radioiodine Therapy Unit as a “No-Care” Ward – A First Experience Report

**DOI:** 10.1055/a-2733-4579

**Published:** 2025-11-24

**Authors:** Martin Freesmeyer, Christian Kühnel, Eike Voigt, Tabea Nikola Schmidt, Philipp Seifert, Falk Gühne, Thomas Winkens

**Affiliations:** 1 Clinic of Nuclear Medicine, Jena University Hospital, Jena, Germany

**Keywords:** Radioiodine Therapy, No-Care, Student assistants, nursing staff shortage

## Abstract

**Aim:**

Nuclear medicine landscape has been changing over the past decade due to the rise of radioligand therapies. However, patients receiving radioiodine therapy for benign thyroid disease still account for approx. one third of the patients on a regular nuclear medicine ward. A substantial part of these patients are hospitalized for radiation protection only and do not require nursing staff. This report aims at describing the implementation of a “no-care” nuclear medicine ward with medical students as staff. We report on the training process, patient and student satisfaction as well as the impact and strengths of this concept.

**Methods:**

A separated nuclear medicine ward (10 beds) was established at a university hospital in Germany. After specific training, two students were assigned per working shift in a regular three-shift-system. Patients were evaluated according to predefined inclusion and exclusion criteria. Patients and students answered two separate surveys, assessing the satisfaction with the concept.

**Results:**

172/319 (53.9%) of the patients met the inclusion criteria. The “no-care” ward was opened six times between April 2024 and June 2025 and the duration was between 10 and 20 days. 101 patients were treated using I-131 sodium iodine, achieving 68.5 DRG relative units. Patient satisfaction survey revealed very high positive response rates. 27 medical students were assigned to the “no-care” ward. The majority of students stated a positive effect on overall medical knowledge and workflow understanding in a hospital.

**Conclusion:**

Using medical students as staff on a “no-care” nuclear medicine ward is feasible and safe. In view of nursing staff shortage, this concept might contribute to adaptive caring in nuclear medicine therapies after careful patient selection.

## Introduction

Nursing staff in nuclear medicine represents an important pillar for radionuclide therapy. The situation in Germany is characterized by strict radiation protection laws, requiring in-patient treatment for most systemically applied therapies in order to handle radioactive excretions and protect surrounding persons from avoidable radiation exposure. The duration of the hospital stay varies from 2 days (most of excess radioactivity has been renally excreted by then) to approx. 3 weeks (until dosage rate limits for safe dismissal are reached). The majority of patients are released from the nuclear medicine ward in less than 4 days of hospitalization. Table 1 summarizes the most frequent indications/diseases. It has to be emphasized that there are long-term in-patient treatments, especially in cases of large goiters and high amounts of therapeutic activity (>1.2 GBq I-131) or metastasized thyroid cancer and very high amounts of therapeutic activity (>5 GBq I-131).

**Table:** **Table 1** Numbers of nuclear medicine in-patient treatment cases in Germany in 2024, according to 1 . * Considering various origins for neuroendocrine tumors, OPS (Operationen- und Prozedurenschlüssel) was chosen instead of DRG in order to retrieve correct data. DRG: Diagnosis related group, rhTSH = recombinant human thyroidea-stimulating hormone, HCC = hepatocellular carcinoma, CCC= cholangiocellular carcinoma, mCRC = metastasized colorectal carcinoma, TARE = transarterial radioembolization, SD = standard deviation.

DRG	Disease	Radiopharmaceutical	Cases 2024	Duration (days; mean ± SD)
**K15E**	Benign thyroid disease	<1.2 GBq I-131	12929	3.1±1.7
**K15D**	Benign thyroid disease	1.2–5.0 GBq I-131	1526	6.0±3.4
		**sum**	**14455**	
**M10B**	Prostate Cancer	7.4 GBq Lu-177-PSMA	8266	2.4±0.9
**K15A**	Thyroid cancer	>5.0 GBq I-131	4265	2.9±1.1
**Z64A, -B**	Thyroid cancer, diagnostic ± rhTSH	0.4 GBq I-131	3529	2.4±0.5
8–530.61,-2*	Neuroendocrine Tumors	7.4 GBq Lu-177-DOTATATE, -TOC	3435	2.6±2.5
**K15C**	Thyroid cancer	1.2–5.0 GBq I-131	2425	3.2±1.8
**H29Z**	HCC, CCC, mCRC	0.5–5 GBq Y-90 Particles, TARE	743	2.8±2.2
		**sum**	**22663**	

Two diametric aspects have shaped the landscape of nuclear medicine treatments over the past decade:

- Nowadays, patients treated on nuclear medicine wards are older, have more comorbidities and present in a worse general shape than 10 years ago. This is attributable to the rise and increasing use of tumor-specific radioligand therapies, especially Lu-177-PSMA (including Pluvicto and patient-individual in-house produced Lu-177-PSMA) and Lu-177-labelled somatostatin analogs (including Lutathera and patient-individual in-house produced Lu-177-DOTATOC/-TATE) as well as emerging radioligand therapies (e.g. Lu-177-FAPI) that have been used as last line in heavily pretreated cancer patients 2 . Regarding commercially available radiopharmaceuticals, a trend towards less heavily pretreated cancer patients has been observed recently. These factors contribute to a high need in professional nursing care including specialized oncology nursing staff with adequate knowledge of patient needs in late-stage cancer as well as psychological and physical robustness.
- Nursing staff has become a scarce resource. As of today, there is a shortage of nurses in hospitals, out-patient care and nursing homes 3 . The demand of additional nurses in hospitals is estimated at 100000 until 2049 4 .

Despite of the increasing morbidity of patients on nuclear medicine wards, it is important to consider that approx. one third of the patients on the nuclear medicine ward consist of rather healthy patients requiring radioiodine therapy of goiter or hyperfunctioning thyroid nodules 1 ( Table 1 ). Typically, these patients are not much affected by the thyroid disease and neither symptoms of hyperthyroidism nor acute side effects of radioiodine therapy require a hospital admission for medical reasons (except rare cases of thyrotoxicosis, severe ophthalmopathy or airway compression) 5 . This group of patients is on the nuclear medicine ward solely for radiation protection reasons and does not require specific nursing staff. Considering the shortage of nursing staff, it seems irrational to allocate highly trained nursing staff to a nuclear medicine ward hosting rather healthy patients.

**Fig. 1:**
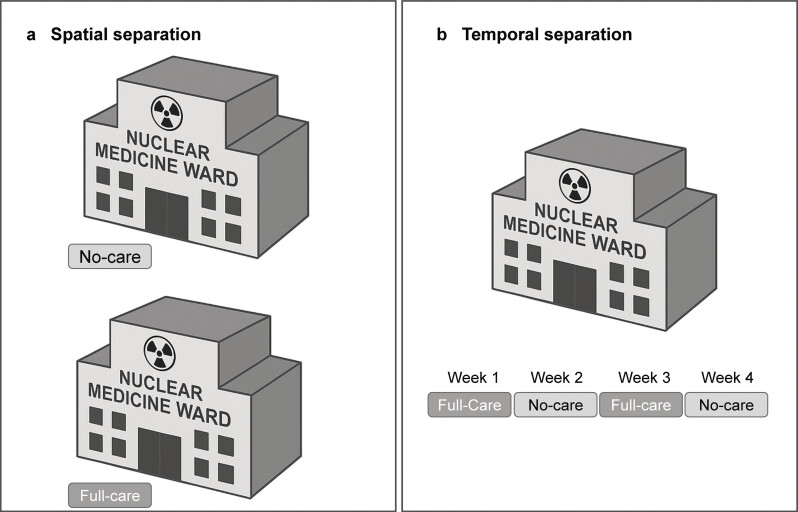
Two concepts of patient separation. **a** ) spatial separation requiring two separated, fully functioning nuclear medicine wards which can be used at the same time. **b** ) temporal separation requiring only one nuclear medicine ward. Patients are scheduled according to their eligibility for a “full-care” or “no-care” week.

Without doubt, it is not constructive to demand a decrease of nursing staff in view of the increasingly challenging group of cancer patients treated in nuclear medicine. But if a spatial or temporal separation of thyroid patients (without requirement of full care; no-care) and cancer patients (full-care) is feasible ( Fig. 1 ), the use of less qualified personnel can be considered for a no-care nuclear medicine ward.

During the past two years we developed a concept of spatial separation for a nuclear medicine ward in a university hospital, successfully dividing patients into two groups (suitable/not suitable for a no-care-ward) and training medical students (student assistants) to carry out tasks to support the patients during the in-patient stay. Here, we report on a) the implementation and training process, b) patient and student satisfaction as assessed by two separate surveys. Lastly, we discuss the impact and strengths as well as limitations of this concept.

## Materials/Methods

### Ethics

All participants of the questionnaire agreed to the use of their data for scientific purposes and provided written informed consent. All investigations were conducted in accordance with the declaration of Helsinki as revised in 2013. The ethical authorization was given by the Ethics Commission of the University Clinic Jena. Date of permission was January 12, 2024, with the registration number 2024-3207.

### Nuclear medicine wards

The concept of spatial separation was implemented in 2024 in a university hospital in Germany. Due to new construction of a substantial part of the entire university hospital, a new nuclear medicine ward hosting 11 patient beds was put into operation in 2017. The formerly used nuclear medicine ward was refurbished in 2010 and is equipped with 7 patient rooms (3× two beds, 4× one bed) and a fully functioning decay installation as required per national legislation. It served as a back-up ward from 2017 to 2024 in times of outage and maintenance of the new nuclear medicine ward, thus technical availability was sustained. The former nuclear medicine ward was chosen for implementation of the no-care-concept (no-care-ward) while operation of the newer nuclear medicine ward (full-care-ward) was maintained at the same time.

### Student assistants

Student assistants were casted for work on the nuclear medicine ward. An invitation email was sent to all medical students regardless of the year of medical school and 34 students replied. Of these, 27 students received training of minimum two 8-hours shifts on the new nuclear medicine ward, familiarizing the students with commonly treated diseases, workflows, documentation processes and radiation protection. Latter was intensified by a qualified instruction by medicine physicist experts.

Per shift, two student assistants were assigned according to previously stated working time preferences. Thus, each day, six students worked on the nuclear medicine ward (early shift 06.00 am – 2.15 pm, late shift 1.45 pm – 10.00 pm, night shift 9.30 pm – 06.15 am), ensuring 24/7 on-site support of the patients. The number of two students per shift was chosen in order to have a backup in case of illness and to reduce errors and overburdening due to one person only on site. Occupational dosimetry was performed according to national legislation using standard dosimeters (LPS-OSL-GD 01 Hp,10 DE-17-M-PTB-0001; Physikalisch Technische Bundesanstalt, Braunschweig, Germany; lower detection limit: 0.1 mSv) which were analyzed retrospectively.

### Patients

Patient groups eligible for the no-care-ward were defined according to specific inclusion and exclusion criteria ( Table 2 ) during a mandatory pretherapeutic radioiodine uptake test which took place 1–10 weeks before in-patient treatment. The final decision was made by a board-certified nuclear medicine specialist. In short, only self-dependent patients requiring radioiodine therapy (RIT) for benign thyroid disease were considered for the no-care-concept. No limits were defined regarding the amount of administered activity or planned duration of the in-patient stay. Upon admission, each patient was seen by a doctor and when giving informed consent regarding radioiodine therapy, was concomitantly instructed regarding their stay on the no-care-ward.

**Table:** **Table 2** Inclusion and exclusion criteria for eligibility of patients with benign thyroid diseases to be treated on a “no-care” nuclear medicine ward. RIT = Radioiodine therapy, NET = neuroendocrine tumor, TARE = transarterial radioembolization, TSH = thyroidea stimulating hormone, ULN = upper limit normal.

	Inclusion criteria	Exclusion criteria
Indication	RIT for Thyroid autonomy Graves disease Goiter	RIT for thyroid cancer Radioligand therapy for Prostate cancer Radioreceptor therapy for NET TARE for liver tumor
Urgency	Not urgent (RIT in < 3 months acceptable)	Urgent (RIT in < 1 month necessary)
Lab values	TSH of any value fT3, fT4 < 1.5 ULN GFR > 50 ml/min Hemoglobin > 7.0 mmol/l	fT3, fT4 > 1.5 ULN GFR < 50 ml/min Hemoglobin < 7.0 mmol/l
Self-assessment, general state	Good, very good	Moderate, poor
Concomitant disease	Allowed: Arterial hypertension Diabetes mellitus (non-insulin dependent) Hypercholesterinemia Smoking habit	Asthma (requiring inhalative therapy) History of Epilepsy Diabetes mellitus (insulin dependent) Psychiatric disorders Incontinency Dementia Alcohol addiction History of coronary heart disease Surgery within the last 6 months Increased risk of falling
Medication	Self-dependent intake of regular oral medication, e.g. Antihypertensives, statins	Inability to prepare and self-administer oral medication Any intravenous, subcutaneous or intramuscular medication Insulin Asthma spray Anticoagulants
Doctor’s judgement	Eligible	Not eligible

### Questionnaires

Patient questionnaires used to assess general patient satisfaction were distributed to the patients on the day before dismissal from the nuclear medicine ward. The questionnaire was identical to the one used in the entire university hospital and consisted of 9 items assessing different topics of satisfaction (structured procedures, medical treatment, nursing, information, nutrition, cleanliness, facilities/equipment) and recommendation (likeliness of return for future treatments, likeliness of recommendation) using a 4-point Likert scale (always, mostly, rarely, never). In the event of dissatisfaction, patients were invited to select multiple subitems regarding desired improvements.

The student questionnaire was developed to assess experience and preparedness, support, time for extra activities, stress and workload as well as benefit for personal development using appropriate Likert scales. 12 items were included in the survey and additional open questions were integrated to give individual feedback. The questionnaire was answered after the first two (of six) opening phases.

## Results

The nuclear medicine ward was opened six times between April 2024 and June 2025 and the duration was three times 14 days, two times 10 days and one time 20 days (82 days total). Regarding the capacity of 10 beds, this is equivalent to 820 bed*days. During this period, 101 patients were treated for benign thyroid disease and 590 bed*days were occupied (72% operating grade). In 17 patients, >1.2 GBq I-131 was administered (DRG: K15D) and in 84 patients <1.2 GBq I-131 was administered (DRG K15E). Patients were released from the nuclear medicine ward when the dosage rate fell below 3.5 µSv/h in 2 meter distance, according to national legislation. In-patient stay was 9.3±3.6 days (median 10 days; range 3–14 days) for K15D and 5.1±3.9 days (median 3 days; range 2–16 days) for K15E, respectively. In total, 68547 DRG relative units (DRG Relativgewichte) were achieved which is equivalent to approx. 295000 Euros revenue. Regarding personnel costs, student assistants were paid minimum wage (15 Euros/hour) which resulted in approx. 59000 Euros for 82 days, considering the presence of 6 students each day.

Patients were 62.1± 10.4 years old (median 63 years; range 27–84 years) which is significantly younger than the patients treated on the full-care nuclear medicine ward during the time between April 2024 and June 2025 (n=805 patients, 65.5±13.4 years (median 67 years; range 6–93 years); p = 0.0005).

From April 2024 to June 2025, 340 patients underwent a pretherapeutic radioiodine uptake test. In 21 patients, RIT was not recommended (either no treatment necessary or surgery preferred) and of the remaining 319 patients, 172 (53.9%) were found to meet inclusion criteria for RIT on the no-care-ward. 61 patients received RIT on the full-care-ward due to efficiency in operating grade.

**Fig. 2:**
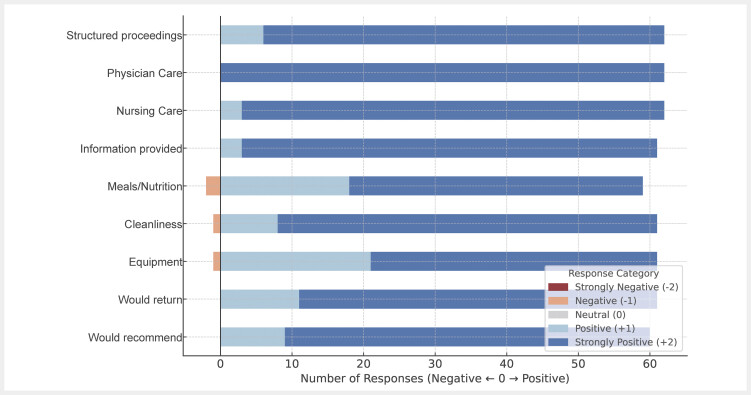
Results of patient questionnaire for nine different items. Positive evaluation is presented as blue bars, negative evaluation as red bars.

The patient questionnaire revealed excellent satisfaction with very high positive response rates and only a few negative answers ( Fig. 2 ). The patients felt well informed and emphasized their strongly positive view on nursing care by the students. Negative ratings were given for meals, cleanliness and general equipment.

**Fig. 3:**
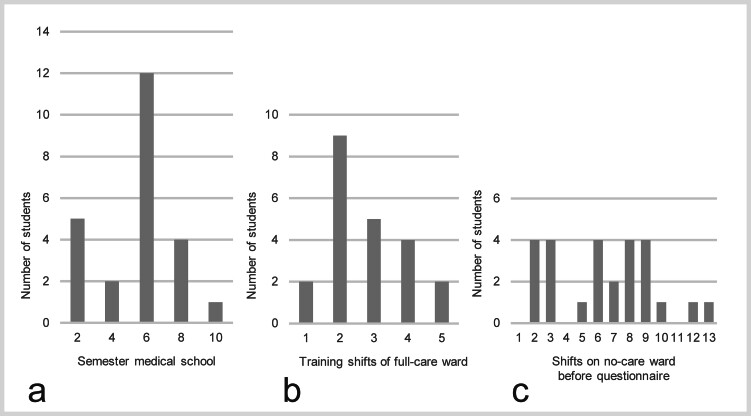
Student assistants’ grade in medical school ( **a** ), training shifts ( **b** ) shifts before answering the questionnaire ( **c** ).

Initially, 34 students replied to the email and 27 decided to start training on the full-care nuclear medicine ward. Students’ age was 21.8 ±1.6 years (median 22; range 19–25) and comprised 22 female, 5 male and 0 diverse. 14/27 students had already been working as student assistants on different wards in other departments of the university clinic. Fig. 3 shows the students’ grade in medical school (a), how many training shifts were taken (b) and how many shifts they worked before answering the questionnaire (c).

Occupational dosimetry revealed minimally detectable values of 0.1 mSv/one month in 2/27 students.

**Fig. 4:**
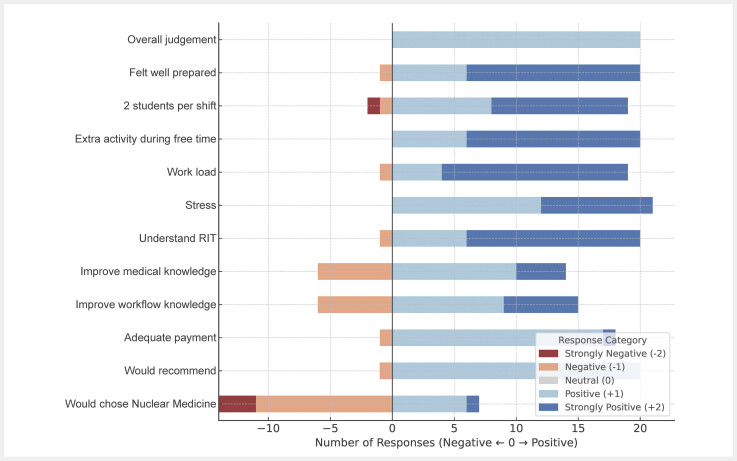
Results of student assistant questionnaire for twelve different items. Positive evaluation is presented as blue bars, negative evaluation as red bars.

Student questionnaire results are shown in Fig. 4 . 24/27 students took part in the questionnaire and 22/27 (81%) completed all questions. The overall judgement was positive, and students felt well prepared. Stress was not perceived. The majority of students stated a positive effect on overall medical knowledge as well as an improvement in their understanding of workflows in a hospital. One student admitted that the work on the nuclear medicine ward convinced him/her to consider nuclear medicine as a specialization after medical school.

## Discussion

Therapeutic nuclear medicine has changed over the past decade 6 . 15 years ago, the majority of patients on a nuclear medicine ward underwent radioiodine therapy for benign thyroid diseases; a therapy that is well-tolerated and not associated with severe acute side-effects. The main reason for these patients for an in-patient stay is radiation protection laws, not medical reasons in the narrow sense. Today, more patients are being treated for cancer on a nuclear medicine ward, often after having received multiple systemic therapies, thus being heavily pretreated and influenced by this pretreatment and/or cancer (and its metastases) itself 2 . This leads to an increasing demand for specialized nuclear medicine nursing staff capable of adequately taking care of the patients 7 8 . Shortage of specialized nursing staff represents a major challenge for the German healthcare system in general and of course, this also applies for nuclear medicine nursing staff 9 .

However, albeit clear increase of cancer patients on nuclear medicine wards 2 and decreasing prevalence of thyroid diseases 10 11 , including conditions in need of therapy, it has to be remembered that approx. one third of patients on nuclear medicine wards are patients receiving a radioiodine therapy for benign thyroid disease. This is also a trend observed in other countries, e.g. the United Kingdom 12 .

The current report shows that the concept of a no-care-ward is feasible in selected patients. Considering the implementation of this concept into clinical workflow, this group of patients represents an almost ideal clientele involving the following advantages: All types of RIT are planned and there are no emergency admissions to the hospital. All patients require a pretherapeutic radioiodine uptake test which is usually performed in an out-patient setting. Thus, it is possible to screen the patients according to their eligibility.

### Student assistants

Student assistants consistently reported positive experiences during their work on the no-care-ward. This is on one hand attributable to low requirements/stress and high gratification (positive feedback, salary) and on the other hand the benefit of gathering experience before finishing medical school. The detailed documentation a nurse has to do, close communication with patients, self-responsible handling of situations (e.g. contaminated food tray, missing supplies, patients’ worries during therapy) and patient-centered structured work are competences that are not primarily taught in medical school. The currently proposed concept is similar to training wards which have been implemented in medical schools in Germany over the past decade and established in Scandinavian countries for more than 20 years 13 14 . Numerous publications describe the positive learning environment and effectiveness as well as interdisciplinary collaboration and communication 15 . Interdisciplinary training wards hosting medical students as well as nursing apprentices and other professions have been found to greatly enhance interprofessional comprehension and insight 16 . Most of the concepts rely on close supervision, briefing and de-briefing meetings in order to analyze workflows and situations 16 . Although every student on the no-care-ward was encouraged to ask questions in case of uncertainty or ambiguity about certain situations and doctors, experienced nurses as well as medicine physicist experts provided close support. However, a structured supervision was not implemented which represents a limitation.

### Patients

Patient safety was ensured at all times and patient questionnaires revealed almost exclusively positive evaluation. No critical incident was reported. This met the expectations the authors had before implementing the no-care-concept and reflects the nature of RIT in patients with benign thyroid diseases. However, the very positive result is likely to be biased, as patients were aware of the recent implementation of the no-care-concept and the conversation between doctor and patient was more extensive than a regular pre-treatment consultation. Thus, it was ensured that patients had a positive and benevolent attitude before RIT was started 17 . Additionally, students showed high motivation, knowing that the patients and the clinic staff had high expectations and thus, they put much effort into doing an optimal job. The positive influence of patient-centered care instead of nursing-defined care in hospitals has been well examined 18 . Furthermore, a recent study by Hellinger et al. reported on the safety and patient satisfaction of an interprofessional training ward in visceral surgery, describing the training ward as a safe concept for patients and that satisfaction levels are even higher than on a regular ward 19 . This is in line with our findings and additionally, it has to be emphasized that visceral surgery possesses a much higher potential for adverse events compared to radioiodine therapy.

### Financial aspects

In Germany, reimbursement of hospital costs is based on DRG-system which was introduced in 2000 and modified in 2020 regarding outsourcing of nursing reimbursement (aG-DRG). The goal of this modification was to avoid unjustified overpayment of nursing costs, and it was assumed that separation of payment for medical treatment and nursing effort would lead to a more accurate cost allocation and reimbursement reflecting actual effort (particularly aiming at adequate coverage of actual nursing costs). It was determined that these costs would henceforth be financed by a nursing budget (negotiated by each hospital with the respective providers). aG-DRG-reimbursement of approx. 295000 Euro covers the costs for the student assistants and leaves approx. 236000 Euro for further costs, i.e. radioiodine capsules, maintenance, infrastructure, energy, food, doctors’ salary. Comparing the budget spent on student assistants with regular nursing staff is difficult and needs closer differentiation. To adequately care for the selected patient group, it is not necessary that two experienced nurses are present on a nuclear medicine ward 24/7. It is common practice to provide two nurses during early shift and one nurse during late and night shift, respectively. Given a median salary of nurses of 4108 Euros/month 3 , the comparable staff budget for experienced nurses calculates to 62000 Euro for 82 days and is therefore slightly higher than the budget spent on students. Considering cost-effectiveness of similar concepts, it has been reported that interdisciplinary training wards in Germany during COVID-19 pandemia contributed to the health system in a cost-effective way 20 . Despite a trend towards minor staff expenditure, it must be kept in mind that this is not the main reason for establishing a concept like this. The primary motivation is the increasing shortage of experienced and professional nurses and assistance staff.

### Limitations

This report has several limitations lowering the overall impact. First, it is a single center experience, that has been developed over a longer period of time and that has been adjusted. The transferability to other nuclear medicine wards might be limited due to a different infrastructure (this concept was only possible because of a second, fully functioning nuclear medicine ward). A concept of temporal separation of a full-care and a no-care-ward is shown in Fig. 1 . Second, positive evaluation by patients and students was biased by a dedicated attitude of doctors, nurses and medicine physics experts who provided close support to the students. Patients were carefully informed about the concept and thus, this also contributed to a positive attitude towards their hospitalization.

## Conclusion

We present a feasible and safe concept of using medical students instead of nursing staff on a nuclear medicine ward. A selected group of patients without co-morbidities requiring radioiodine therapy for benign thyroid diseases are highly satisfied with the students’ performance. Students rate the work as predominantly positive as they have the opportunity to gather experience, receive adequate salary and have time to study during low-stress periods. This concept might even contribute to recruiting medical doctors by providing deep insights into nuclear medicine.
